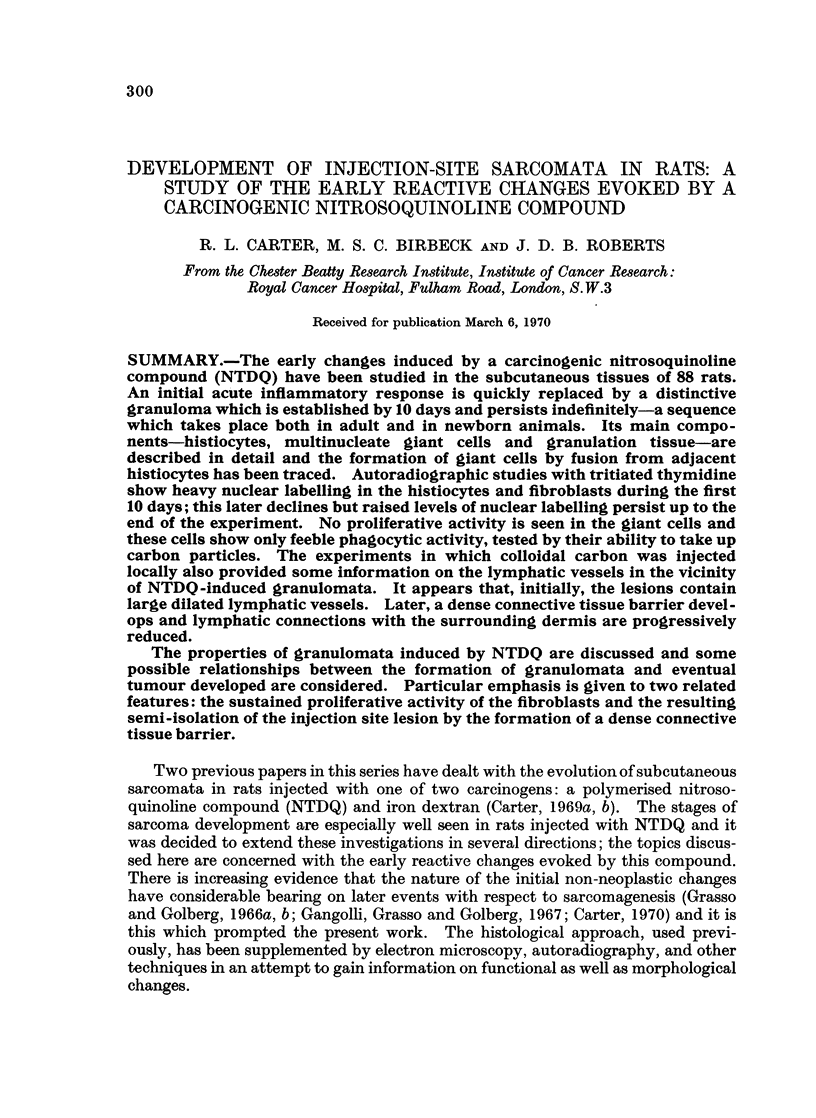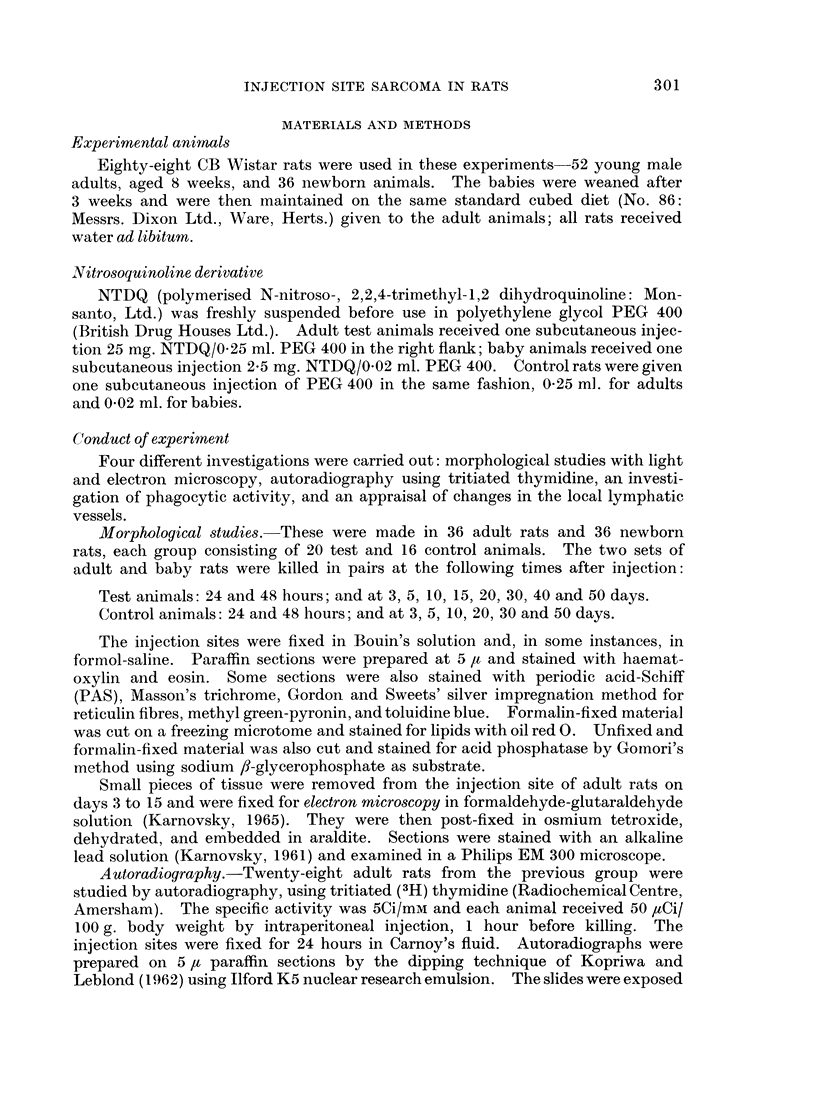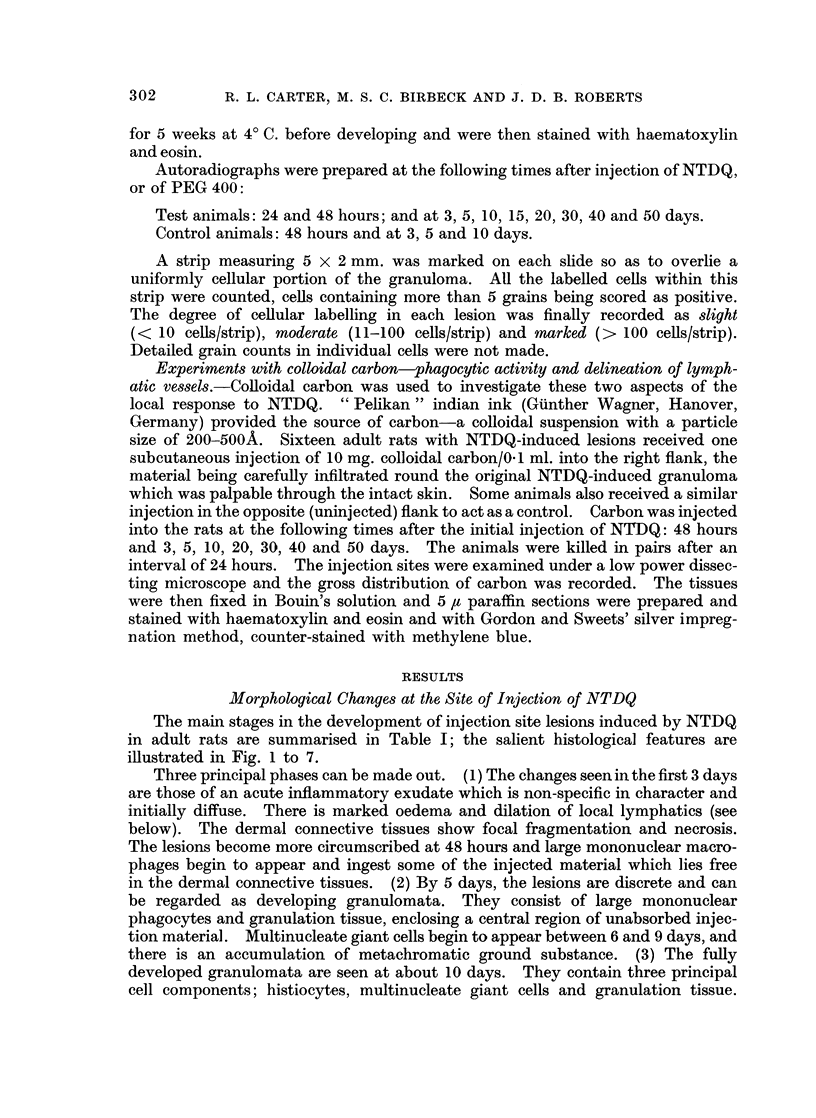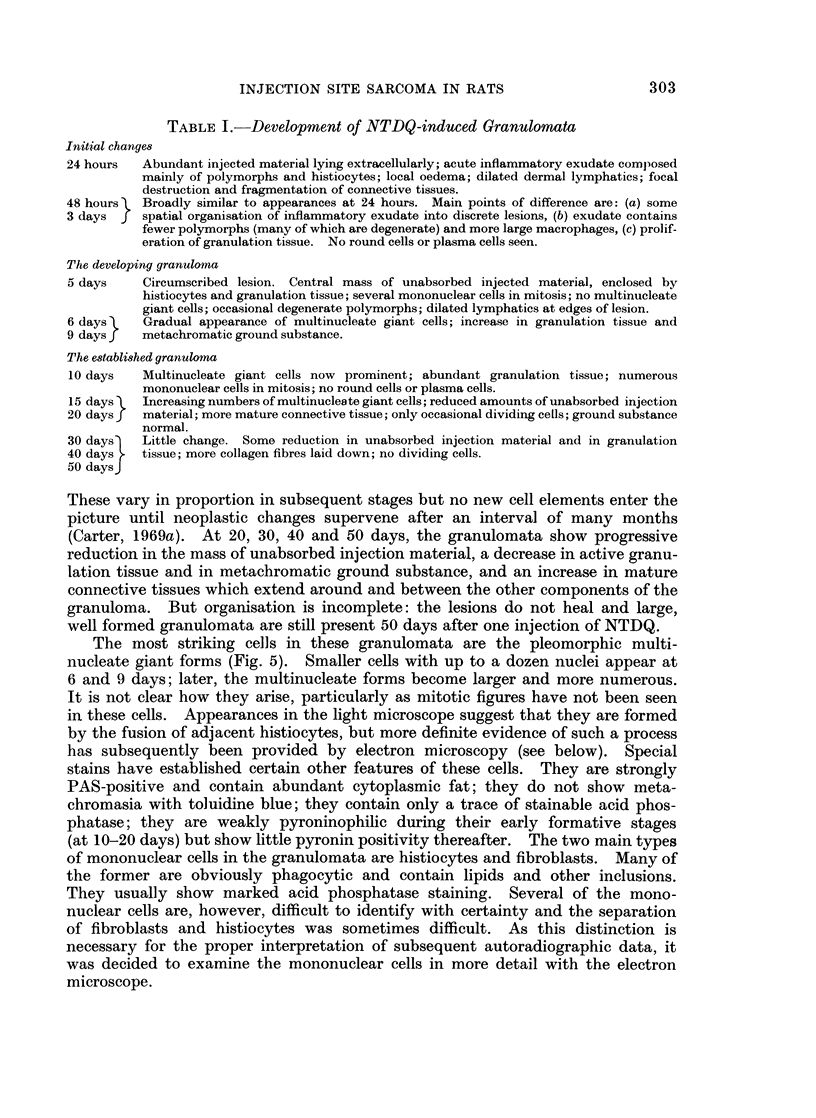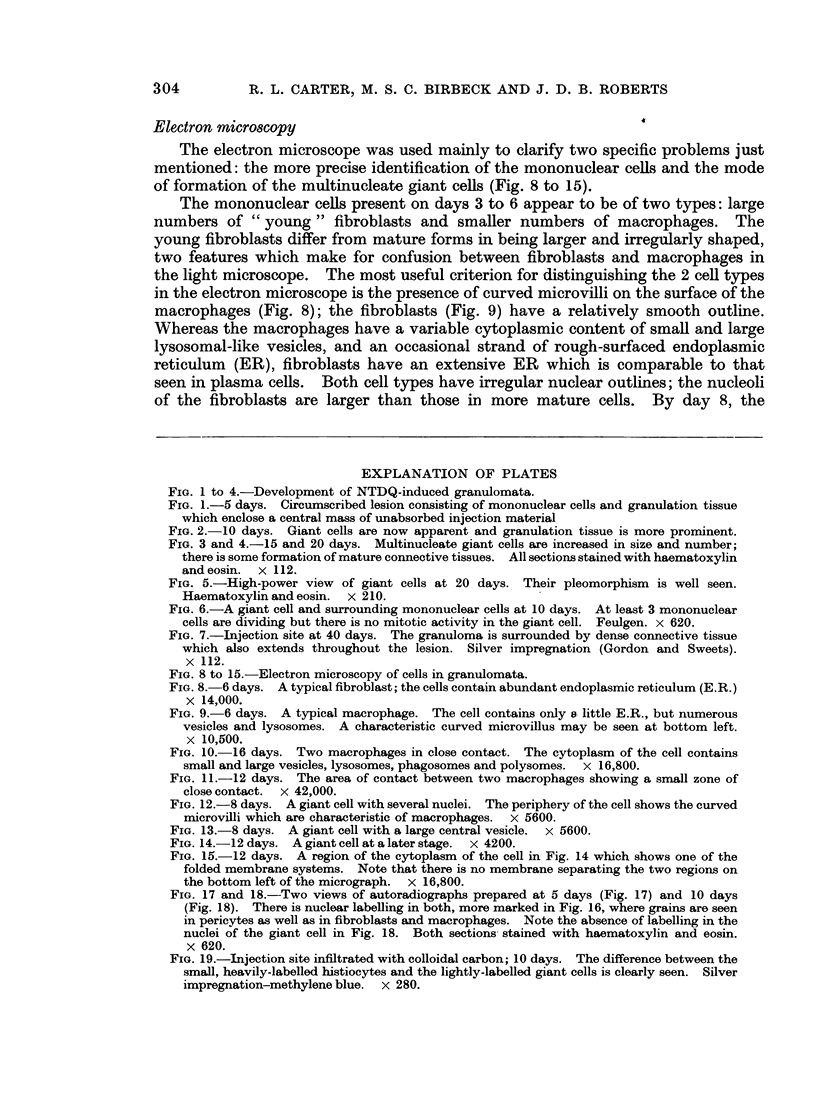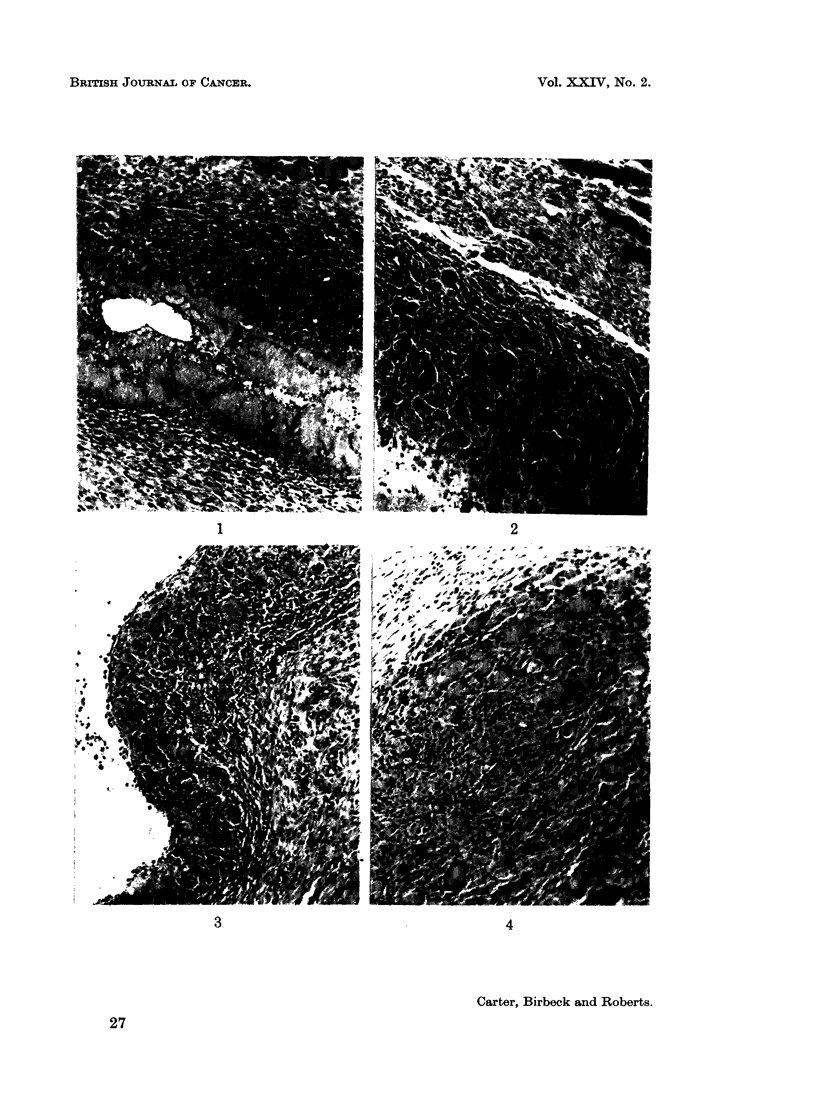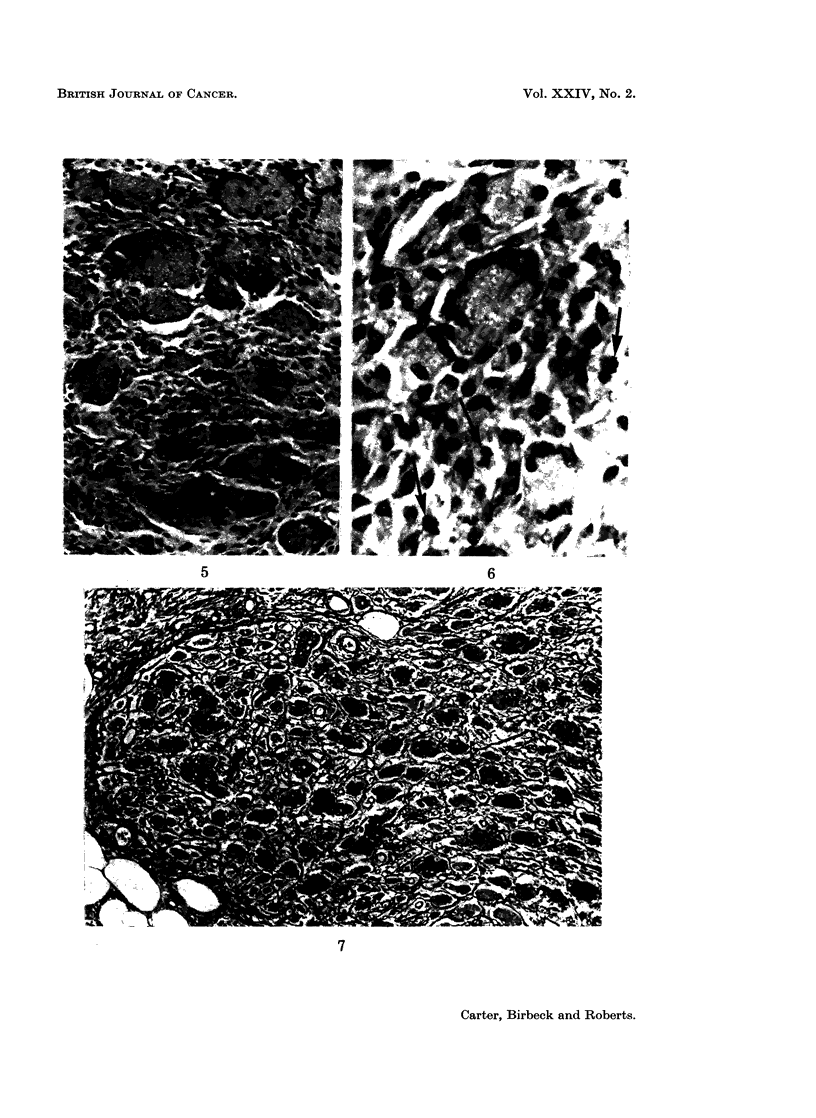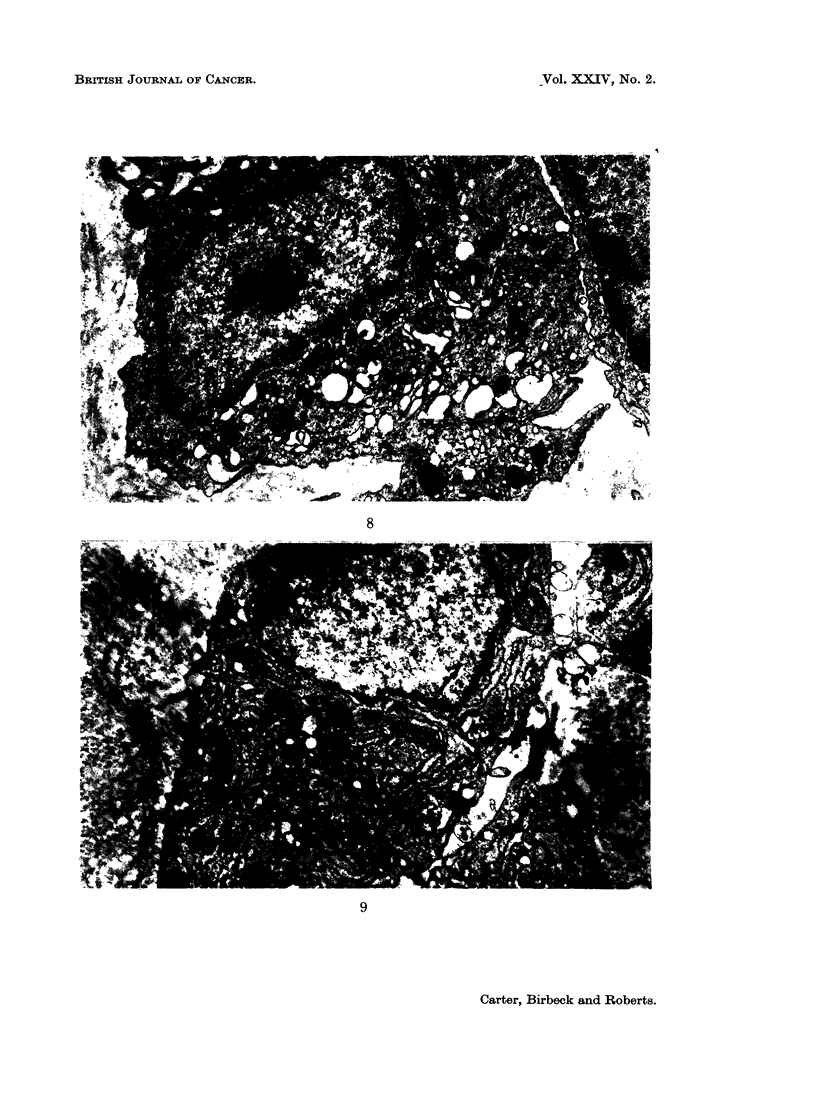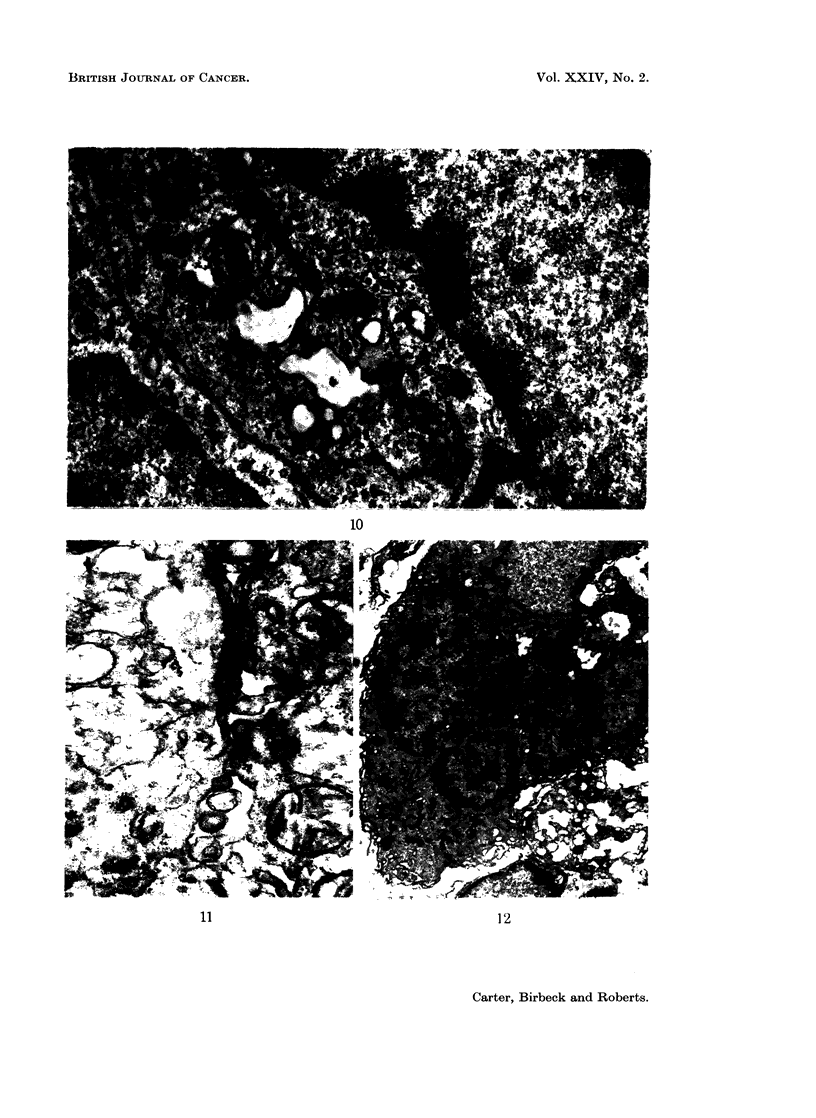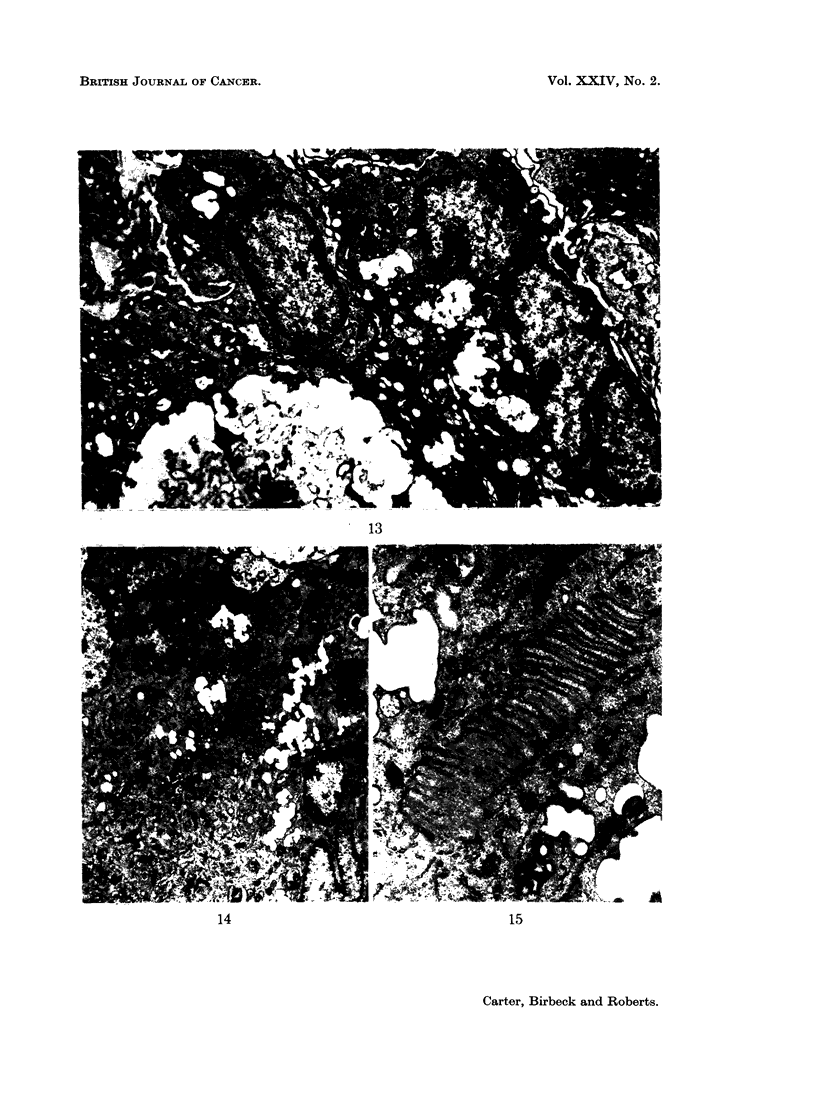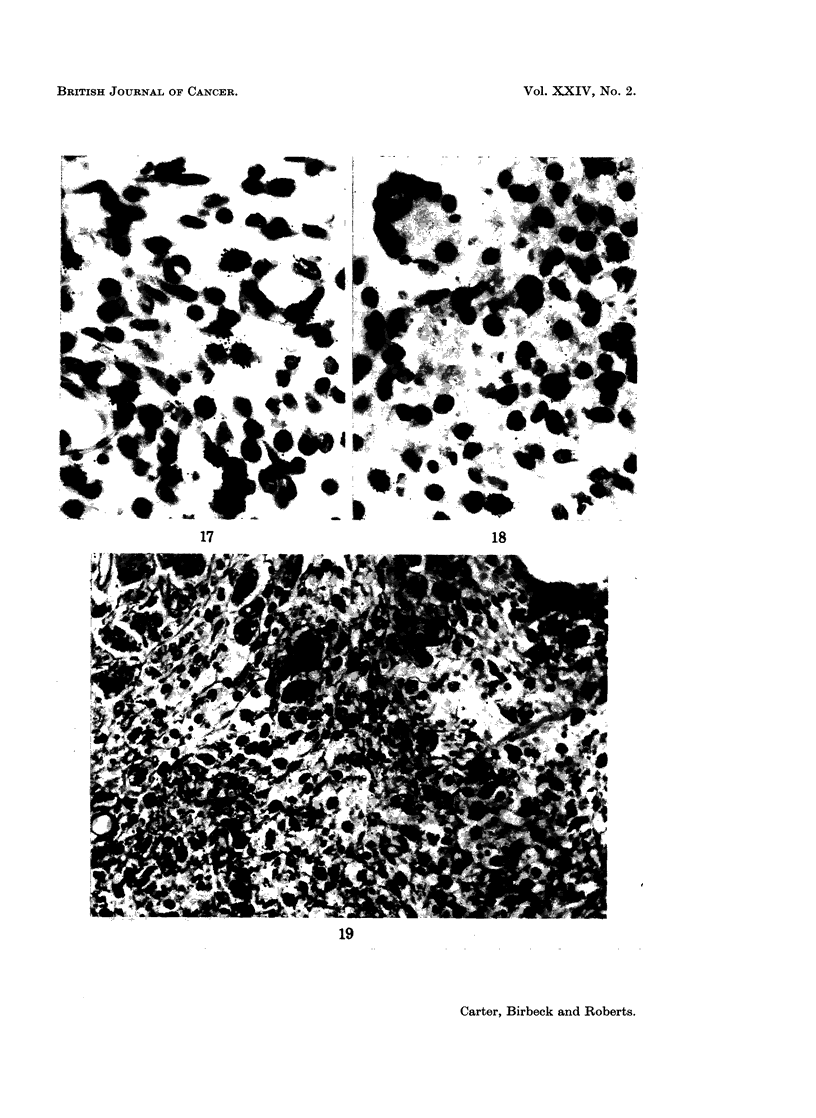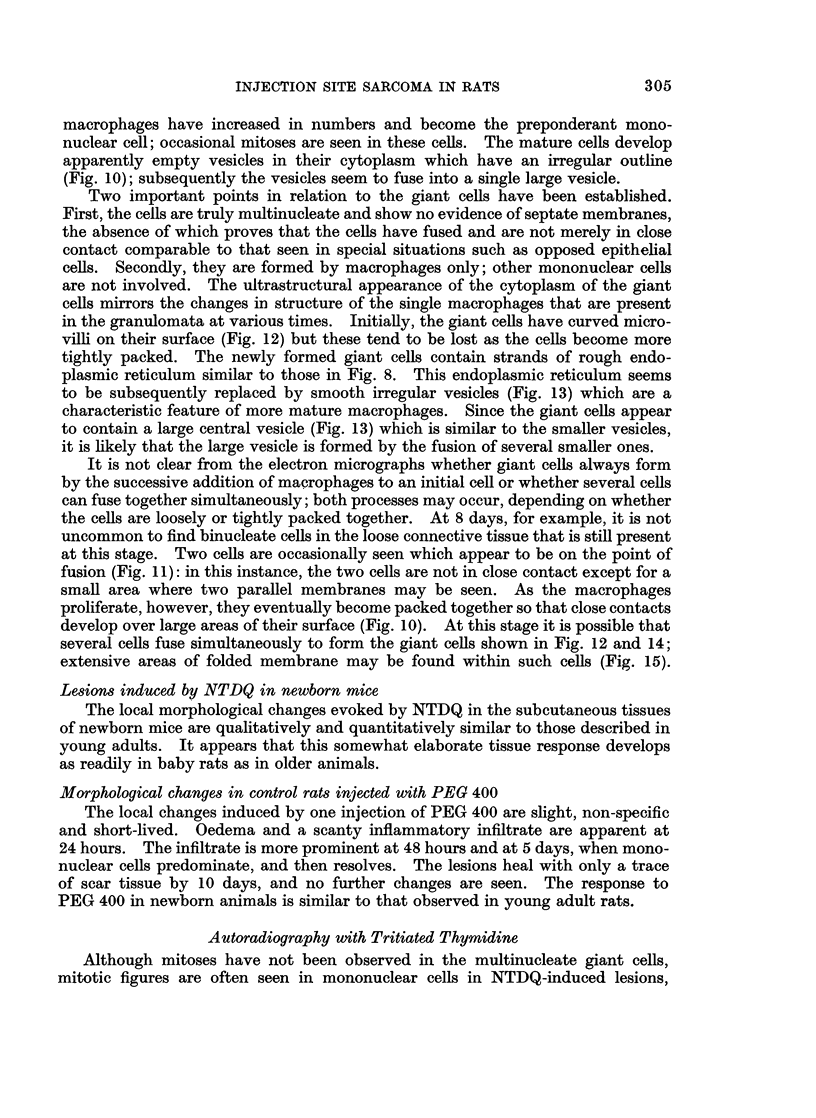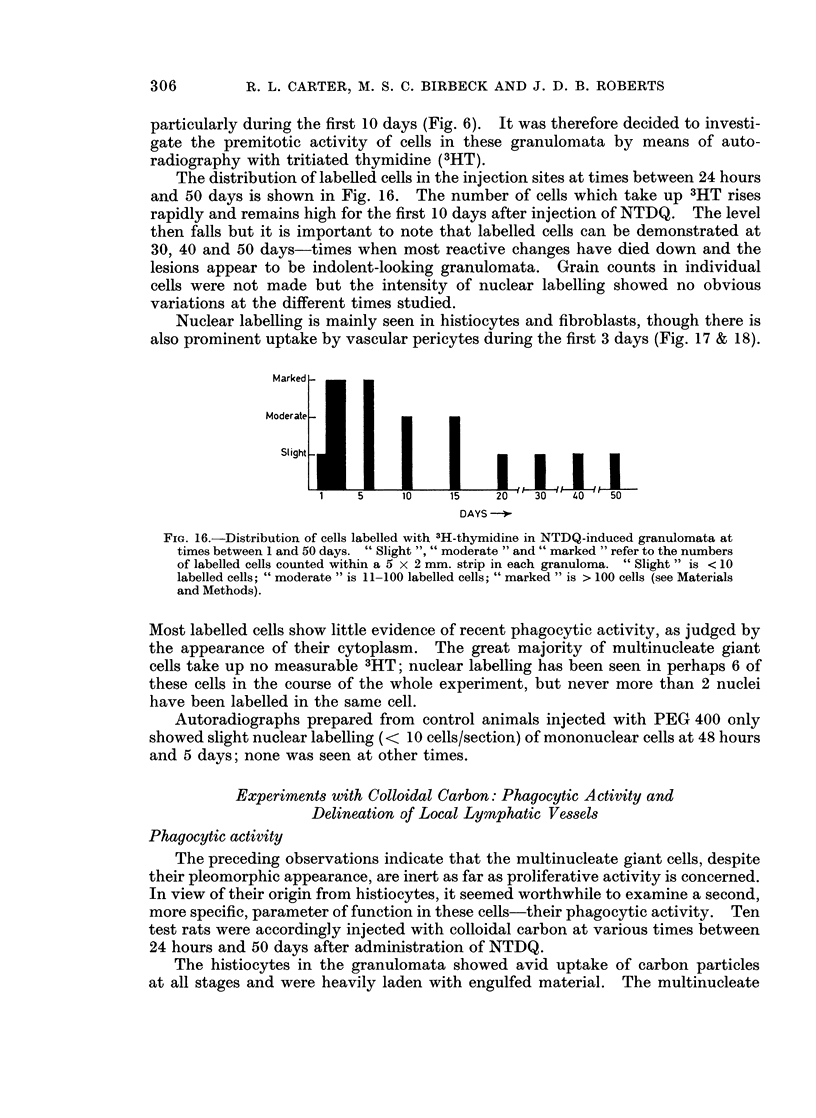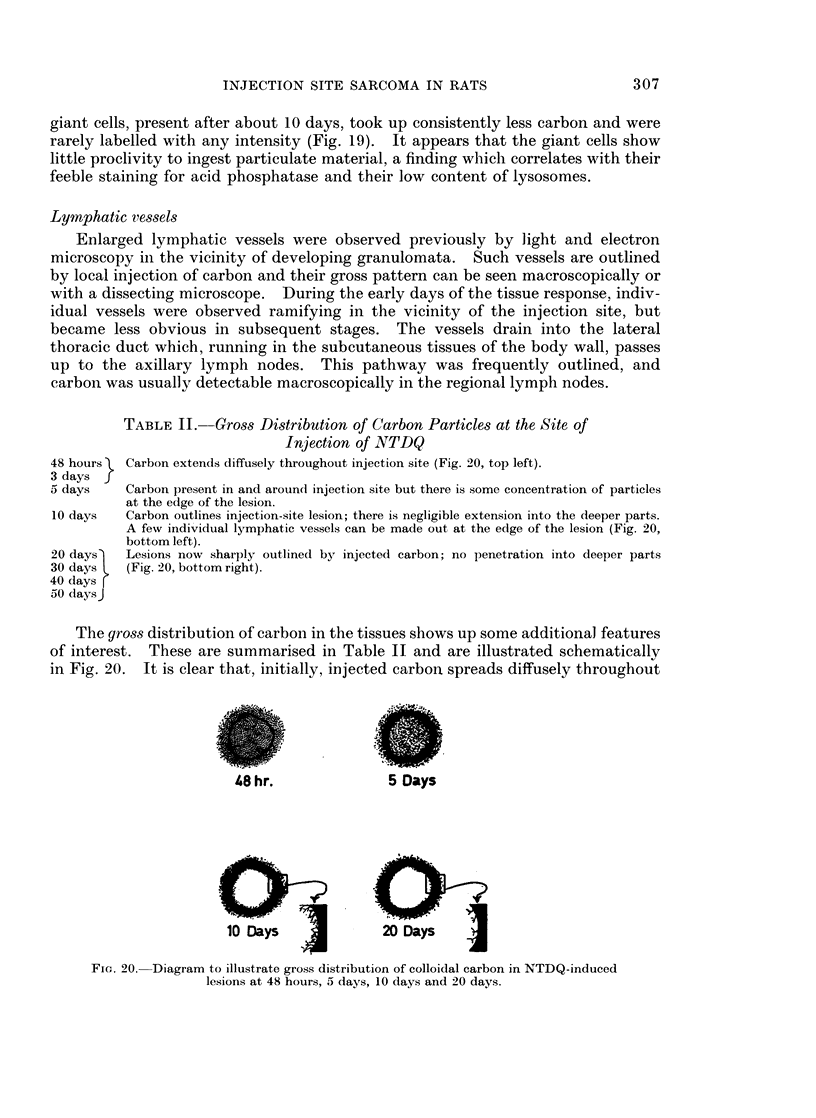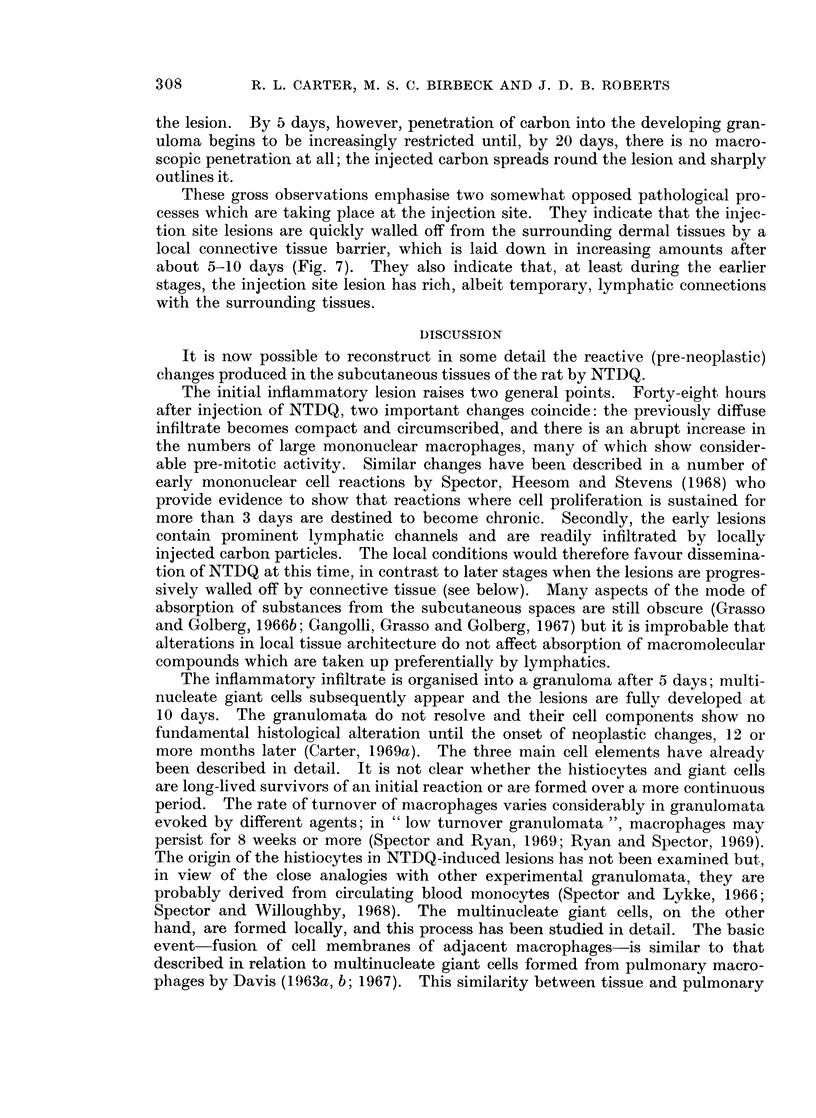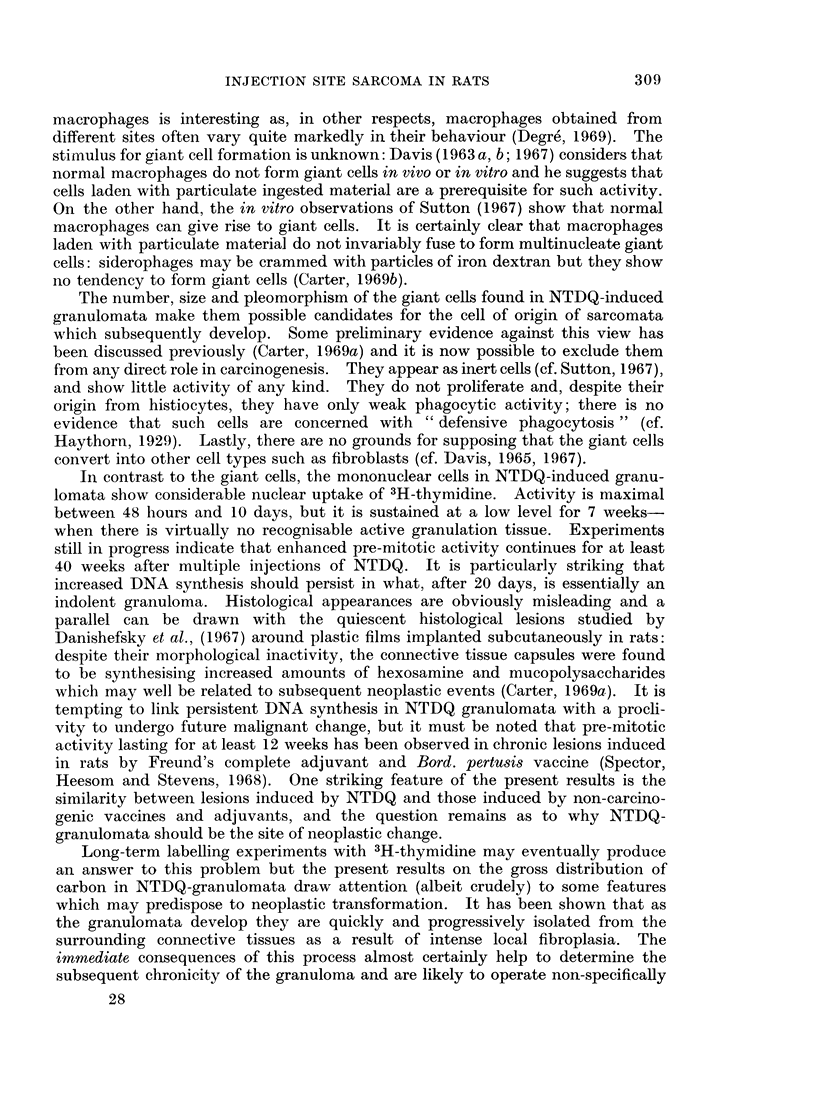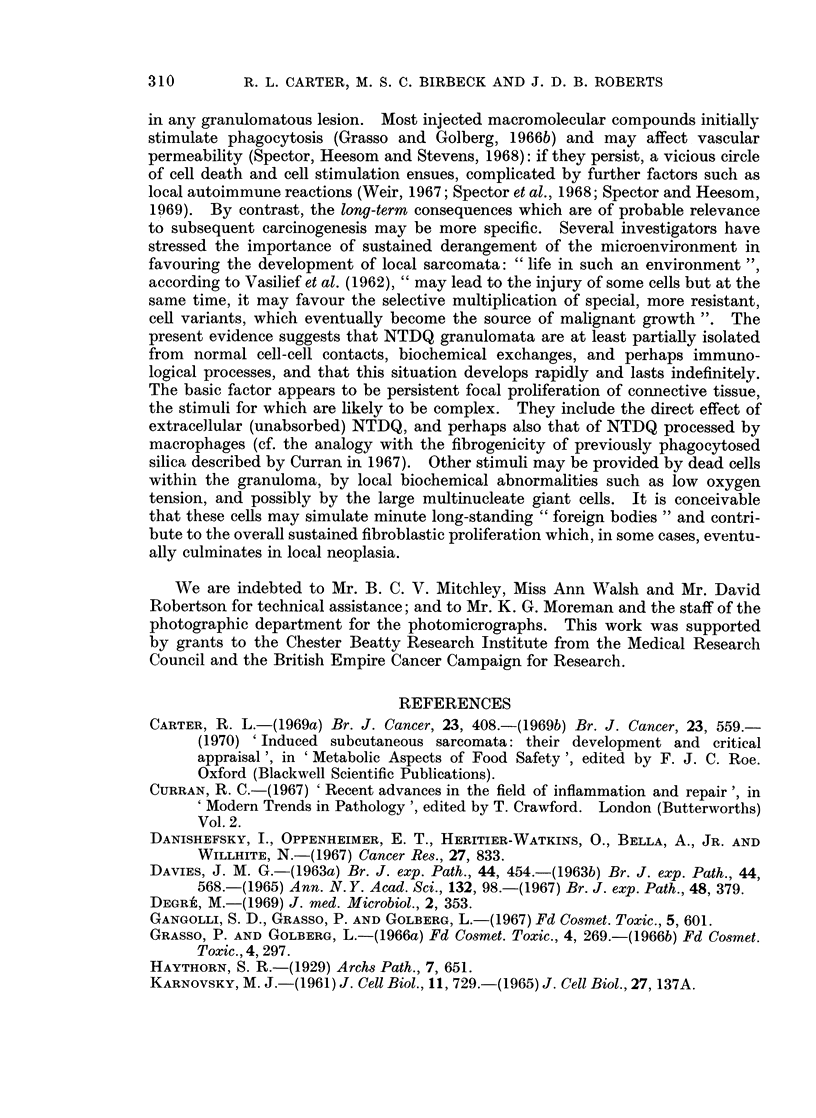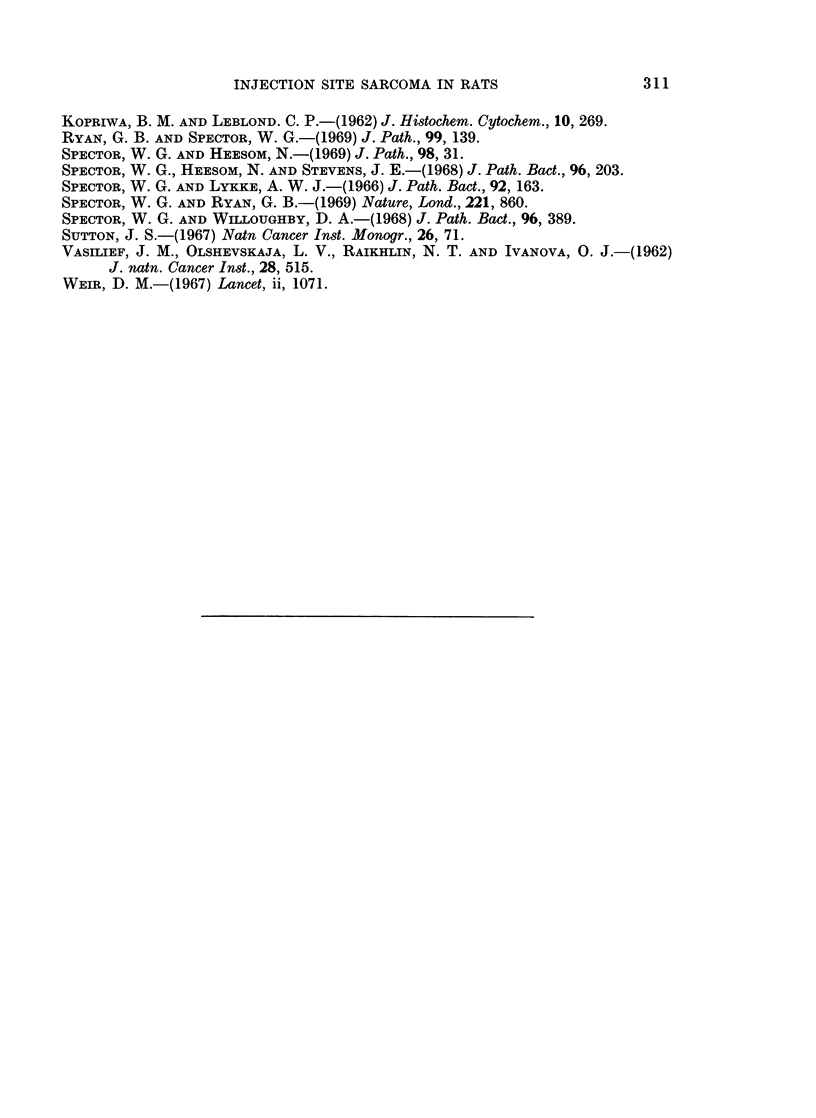# Development of Injection-site Sarcomata in Rats: A Study of the Early Reactive Changes Evoked by a Carcinogenic Nitrosoquinoline Compound

**DOI:** 10.1038/bjc.1970.35

**Published:** 1970-06

**Authors:** R. L. Carter, M. S. C. Birbeck, J. D. B. Roberts

## Abstract

**Images:**


### DEVELOPMENT OF INJECTION-SITE SARCOMATA IN RATS: A STUDY OF THE EARLY REACTIVE CHANGES EVOKED BY A CARCINOGENIC NITROSOQUINOLINE COMPOUND

R. L. CARTER, M. S. C. BIRBECK AND J. D. B. ROBERTS

*From the Chester Beatty Research Institute, Institute of Cancer Research: Royal Cancer Hospital, Fulham Road, London, S.W.3*

Received for publication March 6, 1970

**SUMMARY.—The early changes induced by a carcinogenic nitrosoquinoline compound (NTDQ) have been studied in the subcutaneous tissues of 88 rats. An initial acute inflammatory response is quickly replaced by a distinctive granuloma which is established by 10 days and persists indefinitely—a sequence which takes place both in adult and in newborn animals. Its main components—histiocytes, multinucleate giant cells and granulation tissue—are described in detail and the formation of giant cells by fusion from adjacent histiocytes has been traced. Autoradiographic studies with tritiated thymidine show heavy nuclear labelling in the histiocytes and fibroblasts during the first 10 days; this later declines but raised levels of nuclear labelling persist up to the end of the experiment. No proliferative activity is seen in the giant cells and these cells show only feeble phagocytic activity, tested by their ability to take up carbon particles. The experiments in which colloidal carbon was injected locally also provided some information on the lymphatic vessels in the vicinity of NTDQ-induced granulomata. It appears that, initially, the lesions contain large dilated lymphatic vessels. Later, a dense connective tissue barrier develops and lymphatic connections with the surrounding dermis are progressively reduced.**

**The properties of granulomata induced by NTDQ are discussed and some possible relationships between the formation of granulomata and eventual tumour developed are considered. Particular emphasis is given to two related features: the sustained proliferative activity of the fibroblasts and the resulting semi-isolation of the injection site lesion by the formation of a dense connective tissue barrier.**

Two previous papers in this series have dealt with the evolution of subcutaneous sarcomata in rats injected with one of two carcinogens: a polymerised nitrosoquinoline compound (NTDQ) and iron dextran (Carter, 1969*a*, *b*). The stages of sarcoma development are especially well seen in rats injected with NTDQ and it was decided to extend these investigations in several directions; the topics discussed here are concerned with the early reactive changes evoked by this compound. There is increasing evidence that the nature of the initial non-neoplastic changes have considerable bearing on later events with respect to sarcomagenesis (Grasso and Golberg, 1966*a*, *b*; Gangolli, Grasso and Golberg, 1967; Carter, 1970) and it is this which prompted the present work. The histological approach, used previously, has been supplemented by electron microscopy, autoradiography, and other techniques in an attempt to gain information on functional as well as morphological changes.

### MATERIALS AND METHODS

### *Experimental animals*

Eighty-eight CB Wistar rats were used in these experiments—52 young male adults, aged 8 weeks, and 36 newborn animals. The babies were weaned after 3 weeks and were then maintained on the same standard cubed diet (No. 86: Messrs. Dixon Ltd., Ware, Herts.) given to the adult animals; all rats received water *ad libitum.*

### *Nitrosoquinoline derivative*

NTDQ (polymerised N-nitroso-, 2,2,4-trimethyl-1,2 dihydroquinoline: Monsanto, Ltd.) was freshly suspended before use in polyethylene glycol PEG 400 (British Drug Houses Ltd.). Adult test animals received one subcutaneous injection 25 mg. NTDQ/0·25 ml. PEG 400 in the right flank; baby animals received one subcutaneous injection 2·5 mg. NTDQ/0·02 ml. PEG 400. Control rats were given one subcutaneous injection of PEG 400 in the same fashion, 0·25 ml. for adults and 0·02 ml. for babies.

### *Conduct of experiment*

Four different investigations were carried out: morphological studies with light and electron microscopy, autoradiography using tritiated thymidine, an investigation of phagocytic activity, and an appraisal of changes in the local lymphatic vessels.

*Morphological studies.*—These were made in 36 adult rats and 36 newborn rats, each group consisting of 20 test and 16 control animals. The two sets of adult and baby rats were killed in pairs at the following times after injection:

Test animals: 24 and 48 hours; and at 3, 5, 10, 15, 20, 30, 40 and 50 days.
Control animals: 24 and 48 hours; and at 3, 5, 10, 20, 30 and 50 days.

The injection sites were fixed in Bouin's solution and, in some instances, in formol-saline. Paraffin sections were prepared at 5 $\mu$ and stained with haematoxylin and eosin. Some sections were also stained with periodic acid-Schiff (PAS), Masson's trichrome, Gordon and Sweets' silver impregnation method for reticulin fibres, methyl green-pyronin, and toluidine blue. Formalin-fixed material was cut on a freezing microtome and stained for lipids with oil red O. Unfixed and formalin-fixed material was also cut and stained for acid phosphatase by Gomori's method using sodium $\beta$-glycerophosphate as substrate.

Small pieces of tissue were removed from the injection site of adult rats on days 3 to 15 and were fixed for *electron microscopy* in formaldehyde-glutaraldehyde solution (Karnovsky, 1965). They were then post-fixed in osmium tetroxide, dehydrated, and embedded in araldite. Sections were stained with an alkaline lead solution (Karnovsky, 1961) and examined in a Philips EM 300 microscope.

*Autoradiography.*—Twenty-eight adult rats from the previous group were studied by autoradiography, using tritiated ($^3$H) thymidine (Radiochemical Centre, Amersham). The specific activity was 5Ci/mM and each animal received 50 $\mu$Ci/100 g. body weight by intraperitoneal injection, 1 hour before killing. The injection sites were fixed for 24 hours in Carnoy's fluid. Autoradiographs were prepared on 5 $\mu$ paraffin sections by the dipping technique of Kopriwa and Leblond (1962) using Ilford K5 nuclear research emulsion. The slides were exposed for 5 weeks at 4° C. before developing and were then stained with haematoxylin and eosin.

Autoradiographs were prepared at the following times after injection of NTDQ, or of PEG 400:

Test animals: 24 and 48 hours; and at 3, 5, 10, 15, 20, 30, 40 and 50 days.
Control animals: 48 hours and at 3, 5 and 10 days.

A strip measuring $5 \times 2$ mm. was marked on each slide so as to overlie a uniformly cellular portion of the granuloma. All the labelled cells within this strip were counted, cells containing more than 5 grains being scored as positive. The degree of cellular labelling in each lesion was finally recorded as *slight* ($<$ 10 cells/strip), *moderate* (11–100 cells/strip) and *marked* ($>$ 100 cells/strip). Detailed grain counts in individual cells were not made.

*Experiments with colloidal carbon—phagocytic activity and delineation of lymphatic vessels.*—Colloidal carbon was used to investigate these two aspects of the local response to NTDQ. " Pelikan " indian ink (Günther Wagner, Hanover, Germany) provided the source of carbon—a colloidal suspension with a particle size of 200–500Å. Sixteen adult rats with NTDQ-induced lesions received one subcutaneous injection of 10 mg. colloidal carbon/0·1 ml. into the right flank, the material being carefully infiltrated round the original NTDQ-induced granuloma which was palpable through the intact skin. Some animals also received a similar injection in the opposite (uninjected) flank to act as a control. Carbon was injected into the rats at the following times after the initial injection of NTDQ: 48 hours and 3, 5, 10, 20, 30, 40 and 50 days. The animals were killed in pairs after an interval of 24 hours. The injection sites were examined under a low power dissecting microscope and the gross distribution of carbon was recorded. The tissues were then fixed in Bouin's solution and 5 $\mu$ paraffin sections were prepared and stained with haematoxylin and eosin and with Gordon and Sweets' silver impregnation method, counter-stained with methylene blue.

### RESULTS

### *Morphological Changes at the Site of Injection of NTDQ*

The main stages in the development of injection site lesions induced by NTDQ in adult rats are summarised in Table I; the salient histological features are illustrated in Fig. 1 to 7.

Three principal phases can be made out. (1) The changes seen in the first 3 days are those of an acute inflammatory exudate which is non-specific in character and initially diffuse. There is marked oedema and dilation of local lymphatics (see below). The dermal connective tissues show focal fragmentation and necrosis. The lesions become more circumscribed at 48 hours and large mononuclear macrophages begin to appear and ingest some of the injected material which lies free in the dermal connective tissues. (2) By 5 days, the lesions are discrete and can be regarded as developing granulomata. They consist of large mononuclear phagocytes and granulation tissue, enclosing a central region of unabsorbed injection material. Multinucleate giant cells begin to appear between 6 and 9 days, and there is an accumulation of metachromatic ground substance. (3) The fully developed granulomata are seen at about 10 days. They contain three principal cell components; histiocytes, multinucleate giant cells and granulation tissue.

TABLE I.—*Development of NTDQ-induced Granulomata*

| | |
|---|---|
| *Initial changes* | |
| 24 hours | Abundant injected material lying extracellularly; acute inflammatory exudate composed mainly of polymorphs and histiocytes; local oedema; dilated dermal lymphatics; focal destruction and fragmentation of connective tissues. |
| 48 hours }<br>3 days } | Broadly similar to appearances at 24 hours. Main points of difference are: (*a*) some spatial organisation of inflammatory exudate into discrete lesions, (*b*) exudate contains fewer polymorphs (many of which are degenerate) and more large macrophages, (*c*) proliferation of granulation tissue. No round cells or plasma cells seen. |
| *The developing granuloma* | |
| 5 days | Circumscribed lesion. Central mass of unabsorbed injected material, enclosed by histiocytes and granulation tissue; several mononuclear cells in mitosis; no multinucleate giant cells; occasional degenerate polymorphs; dilated lymphatics at edges of lesion. |
| 6 days }<br>9 days } | Gradual appearance of multinucleate giant cells; increase in granulation tissue and metachromatic ground substance. |
| *The established granuloma* | |
| 10 days | Multinucleate giant cells now prominent; abundant granulation tissue; numerous mononuclear cells in mitosis; no round cells or plasma cells. |
| 15 days }<br>20 days } | Increasing numbers of multinucleate giant cells; reduced amounts of unabsorbed injection material; more mature connective tissue; only occasional dividing cells; ground substance normal. |
| 30 days }<br>40 days }<br>50 days } | Little change. Some reduction in unabsorbed injection material and in granulation tissue; more collagen fibres laid down; no dividing cells. |

These vary in proportion in subsequent stages but no new cell elements enter the picture until neoplastic changes supervene after an interval of many months (Carter, 1969*a*). At 20, 30, 40 and 50 days, the granulomata show progressive reduction in the mass of unabsorbed injection material, a decrease in active granulation tissue and in metachromatic ground substance, and an increase in mature connective tissues which extend around and between the other components of the granuloma. But organisation is incomplete: the lesions do not heal and large, well formed granulomata are still present 50 days after one injection of NTDQ.

The most striking cells in these granulomata are the pleomorphic multinucleate giant forms (Fig. 5). Smaller cells with up to a dozen nuclei appear at 6 and 9 days; later, the multinucleate forms become larger and more numerous. It is not clear how they arise, particularly as mitotic figures have not been seen in these cells. Appearances in the light microscope suggest that they are formed by the fusion of adjacent histiocytes, but more definite evidence of such a process has subsequently been provided by electron microscopy (see below). Special stains have established certain other features of these cells. They are strongly PAS-positive and contain abundant cytoplasmic fat; they do not show metachromasia with toluidine blue; they contain only a trace of stainable acid phosphatase; they are weakly pyroninophilic during their early formative stages (at 10–20 days) but show little pyronin positivity thereafter. The two main types of mononuclear cells in the granulomata are histiocytes and fibroblasts. Many of the former are obviously phagocytic and contain lipids and other inclusions. They usually show marked acid phosphatase staining. Several of the mononuclear cells are, however, difficult to identify with certainty and the separation of fibroblasts and histiocytes was sometimes difficult. As this distinction is necessary for the proper interpretation of subsequent autoradiographic data, it was decided to examine the mononuclear cells in more detail with the electron microscope.

*Electron microscopy*

The electron microscope was used mainly to clarify two specific problems just mentioned: the more precise identification of the mononuclear cells and the mode of formation of the multinucleate giant cells (Fig. 8 to 15).

The mononuclear cells present on days 3 to 6 appear to be of two types: large numbers of " young " fibroblasts and smaller numbers of macrophages. The young fibroblasts differ from mature forms in being larger and irregularly shaped, two features which make for confusion between fibroblasts and macrophages in the light microscope. The most useful criterion for distinguishing the 2 cell types in the electron microscope is the presence of curved microvilli on the surface of the macrophages (Fig. 8); the fibroblasts (Fig. 9) have a relatively smooth outline. Whereas the macrophages have a variable cytoplasmic content of small and large lysosomal-like vesicles, and an occasional strand of rough-surfaced endoplasmic reticulum (ER), fibroblasts have an extensive ER which is comparable to that seen in plasma cells. Both cell types have irregular nuclear outlines; the nucleoli of the fibroblasts are larger than those in more mature cells. By day 8, the

---

EXPLANATION OF PLATES

FIG. 1 to 4.—Development of NTDQ-induced granulomata.

FIG. 1.—5 days. Circumscribed lesion consisting of mononuclear cells and granulation tissue which enclose a central mass of unabsorbed injection material

FIG. 2.—10 days. Giant cells are now apparent and granulation tissue is more prominent.

FIG. 3 and 4.—15 and 20 days. Multinucleate giant cells are increased in size and number; there is some formation of mature connective tissues. All sections stained with haematoxylin and eosin. × 112.

FIG. 5.—High-power view of giant cells at 20 days. Their pleomorphism is well seen. Haematoxylin and eosin. × 210.

FIG. 6.—A giant cell and surrounding mononuclear cells at 10 days. At least 3 mononuclear cells are dividing but there is no mitotic activity in the giant cell. Feulgen. × 620.

FIG. 7.—Injection site at 40 days. The granuloma is surrounded by dense connective tissue which also extends throughout the lesion. Silver impregnation (Gordon and Sweets). × 112.

FIG. 8 to 15.—Electron microscopy of cells in granulomata.

FIG. 8.—6 days. A typical fibroblast; the cells contain abundant endoplasmic reticulum (E.R.) × 14,000.

FIG. 9.—6 days. A typical macrophage. The cell contains only a little E.R., but numerous vesicles and lysosomes. A characteristic curved microvillus may be seen at bottom left. × 10,500.

FIG. 10.—16 days. Two macrophages in close contact. The cytoplasm of the cell contains small and large vesicles, lysosomes, phagosomes and polysomes. × 16,800.

FIG. 11.—12 days. The area of contact between two macrophages showing a small zone of close contact. × 42,000.

FIG. 12.—8 days. A giant cell with several nuclei. The periphery of the cell shows the curved microvilli which are characteristic of macrophages. × 5600.

FIG. 13.—8 days. A giant cell with a large central vesicle. × 5600.

FIG. 14.—12 days. A giant cell at a later stage. × 4200.

FIG. 15.—12 days. A region of the cytoplasm of the cell in Fig. 14 which shows one of the folded membrane systems. Note that there is no membrane separating the two regions on the bottom left of the micrograph. × 16,800.

FIG. 17 and 18.—Two views of autoradiographs prepared at 5 days (Fig. 17) and 10 days (Fig. 18). There is nuclear labelling in both, more marked in Fig. 16, where grains are seen in pericytes as well as in fibroblasts and macrophages. Note the absence of labelling in the nuclei of the giant cell in Fig. 18. Both sections stained with haematoxylin and eosin. × 620.

FIG. 19.—Injection site infiltrated with colloidal carbon; 10 days. The difference between the small, heavily-labelled histiocytes and the lightly-labelled giant cells is clearly seen. Silver impregnation–methylene blue. × 280.

1 2 3 4

Carter, Birbeck and Roberts.

5

6

7

8

9

Carter, Birbeck and Roberts.

10

11

12

13

14

15

Carter, Birbeck and Roberts.

17

18

19

Carter, Birbeck and Roberts.

macrophages have increased in numbers and become the preponderant mononuclear cell; occasional mitoses are seen in these cells. The mature cells develop apparently empty vesicles in their cytoplasm which have an irregular outline (Fig. 10); subsequently the vesicles seem to fuse into a single large vesicle.

Two important points in relation to the giant cells have been established. First, the cells are truly multinucleate and show no evidence of septate membranes, the absence of which proves that the cells have fused and are not merely in close contact comparable to that seen in special situations such as opposed epithelial cells. Secondly, they are formed by macrophages only; other mononuclear cells are not involved. The ultrastructural appearance of the cytoplasm of the giant cells mirrors the changes in structure of the single macrophages that are present in the granulomata at various times. Initially, the giant cells have curved microvilli on their surface (Fig. 12) but these tend to be lost as the cells become more tightly packed. The newly formed giant cells contain strands of rough endoplasmic reticulum similar to those in Fig. 8. This endoplasmic reticulum seems to be subsequently replaced by smooth irregular vesicles (Fig. 13) which are a characteristic feature of more mature macrophages. Since the giant cells appear to contain a large central vesicle (Fig. 13) which is similar to the smaller vesicles, it is likely that the large vesicle is formed by the fusion of several smaller ones.

It is not clear from the electron micrographs whether giant cells always form by the successive addition of macrophages to an initial cell or whether several cells can fuse together simultaneously; both processes may occur, depending on whether the cells are loosely or tightly packed together. At 8 days, for example, it is not uncommon to find binucleate cells in the loose connective tissue that is still present at this stage. Two cells are occasionally seen which appear to be on the point of fusion (Fig. 11): in this instance, the two cells are not in close contact except for a small area where two parallel membranes may be seen. As the macrophages proliferate, however, they eventually become packed together so that close contacts develop over large areas of their surface (Fig. 10). At this stage it is possible that several cells fuse simultaneously to form the giant cells shown in Fig. 12 and 14; extensive areas of folded membrane may be found within such cells (Fig. 15).

*Lesions induced by NTDQ in newborn mice*

The local morphological changes evoked by NTDQ in the subcutaneous tissues of newborn mice are qualitatively and quantitatively similar to those described in young adults. It appears that this somewhat elaborate tissue response develops as readily in baby rats as in older animals.

*Morphological changes in control rats injected with PEG* 400

The local changes induced by one injection of PEG 400 are slight, non-specific and short-lived. Oedema and a scanty inflammatory infiltrate are apparent at 24 hours. The infiltrate is more prominent at 48 hours and at 5 days, when mononuclear cells predominate, and then resolves. The lesions heal with only a trace of scar tissue by 10 days, and no further changes are seen. The response to PEG 400 in newborn animals is similar to that observed in young adult rats.

### *Autoradiography with Tritiated Thymidine*

Although mitoses have not been observed in the multinucleate giant cells, mitotic figures are often seen in mononuclear cells in NTDQ-induced lesions, particularly during the first 10 days (Fig. 6). It was therefore decided to investigate the premitotic activity of cells in these granulomata by means of autoradiography with tritiated thymidine ($^3$HT).

The distribution of labelled cells in the injection sites at times between 24 hours and 50 days is shown in Fig. 16. The number of cells which take up $^3$HT rises rapidly and remains high for the first 10 days after injection of NTDQ. The level then falls but it is important to note that labelled cells can be demonstrated at 30, 40 and 50 days—times when most reactive changes have died down and the lesions appear to be indolent-looking granulomata. Grain counts in individual cells were not made but the intensity of nuclear labelling showed no obvious variations at the different times studied.

Nuclear labelling is mainly seen in histiocytes and fibroblasts, though there is also prominent uptake by vascular pericytes during the first 3 days (Fig. 17 & 18).

Fig. 16.—Distribution of cells labelled with $^3$H-thymidine in NTDQ-induced granulomata at times between 1 and 50 days. " Slight ", " moderate " and " marked " refer to the numbers of labelled cells counted within a 5 × 2 mm. strip in each granuloma. " Slight " is < 10 labelled cells; " moderate " is 11–100 labelled cells; " marked " is > 100 cells (see Materials and Methods).

Most labelled cells show little evidence of recent phagocytic activity, as judged by the appearance of their cytoplasm. The great majority of multinucleate giant cells take up no measurable $^3$HT; nuclear labelling has been seen in perhaps 6 of these cells in the course of the whole experiment, but never more than 2 nuclei have been labelled in the same cell.

Autoradiographs prepared from control animals injected with PEG 400 only showed slight nuclear labelling (< 10 cells/section) of mononuclear cells at 48 hours and 5 days; none was seen at other times.

### *Experiments with Colloidal Carbon: Phagocytic Activity and Delineation of Local Lymphatic Vessels*

### *Phagocytic activity*

The preceding observations indicate that the multinucleate giant cells, despite their pleomorphic appearance, are inert as far as proliferative activity is concerned. In view of their origin from histiocytes, it seemed worthwhile to examine a second, more specific, parameter of function in these cells—their phagocytic activity. Ten test rats were accordingly injected with colloidal carbon at various times between 24 hours and 50 days after administration of NTDQ.

The histiocytes in the granulomata showed avid uptake of carbon particles at all stages and were heavily laden with engulfed material. The multinucleate giant cells, present after about 10 days, took up consistently less carbon and were rarely labelled with any intensity (Fig. 19). It appears that the giant cells show little proclivity to ingest particulate material, a finding which correlates with their feeble staining for acid phosphatase and their low content of lysosomes.

*Lymphatic vessels*

Enlarged lymphatic vessels were observed previously by light and electron microscopy in the vicinity of developing granulomata. Such vessels are outlined by local injection of carbon and their gross pattern can be seen macroscopically or with a dissecting microscope. During the early days of the tissue response, individual vessels were observed ramifying in the vicinity of the injection site, but became less obvious in subsequent stages. The vessels drain into the lateral thoracic duct which, running in the subcutaneous tissues of the body wall, passes up to the axillary lymph nodes. This pathway was frequently outlined, and carbon was usually detectable macroscopically in the regional lymph nodes.

TABLE II.—*Gross Distribution of Carbon Particles at the Site of Injection of NTDQ*

| | |
|---|---|
| 48 hours, 3 days | Carbon extends diffusely throughout injection site (Fig. 20, top left). |
| 5 days | Carbon present in and around injection site but there is some concentration of particles at the edge of the lesion. |
| 10 days | Carbon outlines injection-site lesion; there is negligible extension into the deeper parts. A few individual lymphatic vessels can be made out at the edge of the lesion (Fig. 20, bottom left). |
| 20 days, 30 days, 40 days, 50 days | Lesions now sharply outlined by injected carbon; no penetration into deeper parts (Fig. 20, bottom right). |

The *gross* distribution of carbon in the tissues shows up some additional features of interest. These are summarised in Table II and are illustrated schematically in Fig. 20. It is clear that, initially, injected carbon spreads diffusely throughout

FIG. 20.—Diagram to illustrate gross distribution of colloidal carbon in NTDQ-induced lesions at 48 hours, 5 days, 10 days and 20 days.

the lesion. By 5 days, however, penetration of carbon into the developing granuloma begins to be increasingly restricted until, by 20 days, there is no macroscopic penetration at all; the injected carbon spreads round the lesion and sharply outlines it.

These gross observations emphasise two somewhat opposed pathological processes which are taking place at the injection site. They indicate that the injection site lesions are quickly walled off from the surrounding dermal tissues by a local connective tissue barrier, which is laid down in increasing amounts after about 5–10 days (Fig. 7). They also indicate that, at least during the earlier stages, the injection site lesion has rich, albeit temporary, lymphatic connections with the surrounding tissues.

### DISCUSSION

It is now possible to reconstruct in some detail the reactive (pre-neoplastic) changes produced in the subcutaneous tissues of the rat by NTDQ.

The initial inflammatory lesion raises two general points. Forty-eight hours after injection of NTDQ, two important changes coincide: the previously diffuse infiltrate becomes compact and circumscribed, and there is an abrupt increase in the numbers of large mononuclear macrophages, many of which show considerable pre-mitotic activity. Similar changes have been described in a number of early mononuclear cell reactions by Spector, Heesom and Stevens (1968) who provide evidence to show that reactions where cell proliferation is sustained for more than 3 days are destined to become chronic. Secondly, the early lesions contain prominent lymphatic channels and are readily infiltrated by locally injected carbon particles. The local conditions would therefore favour dissemination of NTDQ at this time, in contrast to later stages when the lesions are progressively walled off by connective tissue (see below). Many aspects of the mode of absorption of substances from the subcutaneous spaces are still obscure (Grasso and Golberg, 1966*b*; Gangolli, Grasso and Golberg, 1967) but it is improbable that alterations in local tissue architecture do not affect absorption of macromolecular compounds which are taken up preferentially by lymphatics.

The inflammatory infiltrate is organised into a granuloma after 5 days; multinucleate giant cells subsequently appear and the lesions are fully developed at 10 days. The granulomata do not resolve and their cell components show no fundamental histological alteration until the onset of neoplastic changes, 12 or more months later (Carter, 1969*a*). The three main cell elements have already been described in detail. It is not clear whether the histiocytes and giant cells are long-lived survivors of an initial reaction or are formed over a more continuous period. The rate of turnover of macrophages varies considerably in granulomata evoked by different agents; in " low turnover granulomata ", macrophages may persist for 8 weeks or more (Spector and Ryan, 1969; Ryan and Spector, 1969). The origin of the histiocytes in NTDQ-induced lesions has not been examined but, in view of the close analogies with other experimental granulomata, they are probably derived from circulating blood monocytes (Spector and Lykke, 1966; Spector and Willoughby, 1968). The multinucleate giant cells, on the other hand, are formed locally, and this process has been studied in detail. The basic event—fusion of cell membranes of adjacent macrophages—is similar to that described in relation to multinucleate giant cells formed from pulmonary macrophages by Davis (1963*a*, *b*; 1967). This similarity between tissue and pulmonary macrophages is interesting as, in other respects, macrophages obtained from different sites often vary quite markedly in their behaviour (Degré, 1969). The stimulus for giant cell formation is unknown: Davis (1963*a*, *b*; 1967) considers that normal macrophages do not form giant cells *in vivo* or *in vitro* and he suggests that cells laden with particulate ingested material are a prerequisite for such activity. On the other hand, the *in vitro* observations of Sutton (1967) show that normal macrophages can give rise to giant cells. It is certainly clear that macrophages laden with particulate material do not invariably fuse to form multinucleate giant cells: siderophages may be crammed with particles of iron dextran but they show no tendency to form giant cells (Carter, 1969*b*).

The number, size and pleomorphism of the giant cells found in NTDQ-induced granulomata make them possible candidates for the cell of origin of sarcomata which subsequently develop. Some preliminary evidence against this view has been discussed previously (Carter, 1969*a*) and it is now possible to exclude them from any direct role in carcinogenesis. They appear as inert cells (cf. Sutton, 1967), and show little activity of any kind. They do not proliferate and, despite their origin from histiocytes, they have only weak phagocytic activity; there is no evidence that such cells are concerned with "defensive phagocytosis" (cf. Haythorn, 1929). Lastly, there are no grounds for supposing that the giant cells convert into other cell types such as fibroblasts (cf. Davis, 1965, 1967).

In contrast to the giant cells, the mononuclear cells in NTDQ-induced granulomata show considerable nuclear uptake of $^3$H-thymidine. Activity is maximal between 48 hours and 10 days, but it is sustained at a low level for 7 weeks—when there is virtually no recognisable active granulation tissue. Experiments still in progress indicate that enhanced pre-mitotic activity continues for at least 40 weeks after multiple injections of NTDQ. It is particularly striking that increased DNA synthesis should persist in what, after 20 days, is essentially an indolent granuloma. Histological appearances are obviously misleading and a parallel can be drawn with the quiescent histological lesions studied by Danishefsky *et al.*, (1967) around plastic films implanted subcutaneously in rats: despite their morphological inactivity, the connective tissue capsules were found to be synthesising increased amounts of hexosamine and mucopolysaccharides which may well be related to subsequent neoplastic events (Carter, 1969*a*). It is tempting to link persistent DNA synthesis in NTDQ granulomata with a proclivity to undergo future malignant change, but it must be noted that pre-mitotic activity lasting for at least 12 weeks has been observed in chronic lesions induced in rats by Freund's complete adjuvant and *Bord. pertusis* vaccine (Spector, Heesom and Stevens, 1968). One striking feature of the present results is the similarity between lesions induced by NTDQ and those induced by non-carcinogenic vaccines and adjuvants, and the question remains as to why NTDQ-granulomata should be the site of neoplastic change.

Long-term labelling experiments with $^3$H-thymidine may eventually produce an answer to this problem but the present results on the gross distribution of carbon in NTDQ-granulomata draw attention (albeit crudely) to some features which may predispose to neoplastic transformation. It has been shown that as the granulomata develop they are quickly and progressively isolated from the surrounding connective tissues as a result of intense local fibroplasia. The *immediate* consequences of this process almost certainly help to determine the subsequent chronicity of the granuloma and are likely to operate non-specifically in any granulomatous lesion. Most injected macromolecular compounds initially stimulate phagocytosis (Grasso and Golberg, 1966*b*) and may affect vascular permeability (Spector, Heesom and Stevens, 1968): if they persist, a vicious circle of cell death and cell stimulation ensues, complicated by further factors such as local autoimmune reactions (Weir, 1967; Spector *et al.*, 1968; Spector and Heesom, 1969). By contrast, the *long-term* consequences which are of probable relevance to subsequent carcinogenesis may be more specific. Several investigators have stressed the importance of sustained derangement of the microenvironment in favouring the development of local sarcomata: " life in such an environment ", according to Vasilief *et al.* (1962), " may lead to the injury of some cells but at the same time, it may favour the selective multiplication of special, more resistant, cell variants, which eventually become the source of malignant growth ". The present evidence suggests that NTDQ granulomata are at least partially isolated from normal cell-cell contacts, biochemical exchanges, and perhaps immunological processes, and that this situation develops rapidly and lasts indefinitely. The basic factor appears to be persistent focal proliferation of connective tissue, the stimuli for which are likely to be complex. They include the direct effect of extracellular (unabsorbed) NTDQ, and perhaps also that of NTDQ processed by macrophages (cf. the analogy with the fibrogenicity of previously phagocytosed silica described by Curran in 1967). Other stimuli may be provided by dead cells within the granuloma, by local biochemical abnormalities such as low oxygen tension, and possibly by the large multinucleate giant cells. It is conceivable that these cells may simulate minute long-standing " foreign bodies " and contribute to the overall sustained fibroblastic proliferation which, in some cases, eventually culminates in local neoplasia.

We are indebted to Mr. B. C. V. Mitchley, Miss Ann Walsh and Mr. David Robertson for technical assistance; and to Mr. K. G. Moreman and the staff of the photographic department for the photomicrographs. This work was supported by grants to the Chester Beatty Research Institute from the Medical Research Council and the British Empire Cancer Campaign for Research.

### REFERENCES

CARTER, R. L.—(1969*a*) *Br. J. Cancer*, **23**, 408.—(1969*b*) *Br. J. Cancer*, **23**, 559.—(1970) ' Induced subcutaneous sarcomata: their development and critical appraisal ', in ' Metabolic Aspects of Food Safety ', edited by F. J. C. Roe. Oxford (Blackwell Scientific Publications).

CURRAN, R. C.—(1967) ' Recent advances in the field of inflammation and repair ', in ' Modern Trends in Pathology ', edited by T. Crawford. London (Butterworths) Vol. 2.

DANISHEFSKY, I., OPPENHEIMER, E. T., HERITIER-WATKINS, O., BELLA, A., JR. AND WILLHITE, N.—(1967) *Cancer Res.*, **27**, 833.

DAVIES, J. M. G.—(1963*a*) *Br. J. exp. Path.*, **44**, 454.—(1963*b*) *Br. J. exp. Path.*, **44**, 568.—(1965) *Ann. N.Y. Acad. Sci.*, **132**, 98.—(1967) *Br. J. exp. Path.*, **48**, 379.

DEGRÉ, M.—(1969) *J. med. Microbiol.*, **2**, 353.

GANGOLLI, S. D., GRASSO, P. AND GOLBERG, L.—(1967) *Fd Cosmet. Toxic.*, **5**, 601.

GRASSO, P. AND GOLBERG, L.—(1966*a*) *Fd Cosmet. Toxic.*, **4**, 269.—(1966*b*) *Fd Cosmet. Toxic.*, **4**, 297.

HAYTHORN, S. R.—(1929) *Archs Path.*, **7**, 651.

KARNOVSKY, M. J.—(1961) *J. Cell Biol.*, **11**, 729.—(1965) *J. Cell Biol.*, **27**, 137A.

KOPRIWA, B. M. AND LEBLOND. C. P.—(1962) *J. Histochem. Cytochem.*, **10**, 269.
RYAN, G. B. AND SPECTOR, W. G.—(1969) *J. Path.*, **99**, 139.
SPECTOR, W. G. AND HEESOM, N.—(1969) *J. Path.*, **98**, 31.
SPECTOR, W. G., HEESOM, N. AND STEVENS, J. E.—(1968) *J. Path. Bact.*, **96**, 203.
SPECTOR, W. G. AND LYKKE, A. W. J.—(1966) *J. Path. Bact.*, **92**, 163.
SPECTOR, W. G. AND RYAN, G. B.—(1969) *Nature, Lond.*, **221**, 860.
SPECTOR, W. G. AND WILLOUGHBY, D. A.—(1968) *J. Path. Bact.*, **96**, 389.
SUTTON, J. S.—(1967) *Natn Cancer Inst. Monogr.*, **26**, 71.
VASILIEF, J. M., OLSHEVSKAJA, L. V., RAIKHLIN, N. T. AND IVANOVA, O. J.—(1962) *J. natn. Cancer Inst.*, **28**, 515.
WEIR, D. M.—(1967) *Lancet*, ii, 1071.