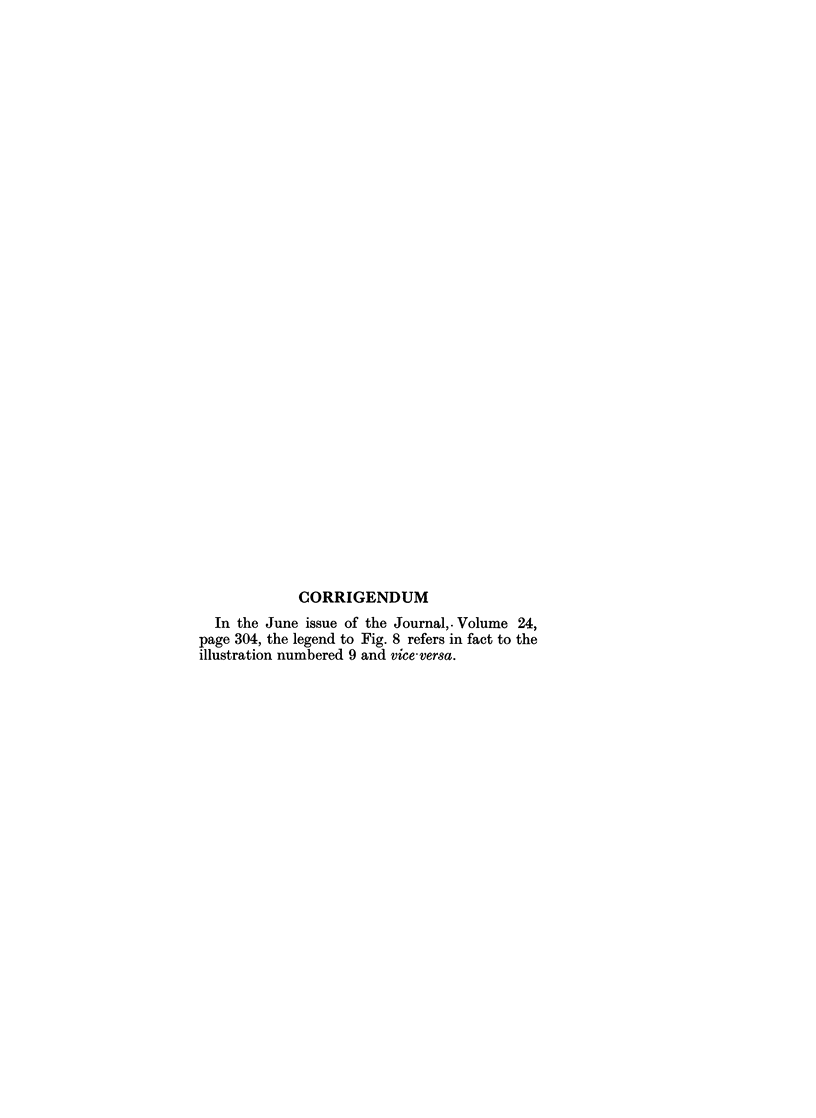# Corrigendum

**Published:** 1970-06

**Authors:** 


### CORRIGENDUM

In the June issue of the Journal, Volume 24, page 304, the legend to Fig. 8 refers in fact to the illustration numbered 9 and *vice versa.*